# Advanced Medical Therapies for Perianal Fistulizing Crohn’s Disease: A Systematic Review of Clinical, Radiological, Surgical, and Composite Outcomes

**DOI:** 10.3390/ph19030417

**Published:** 2026-03-04

**Authors:** Fares Jamal, Tayo Segun-Omosehin, Taylor Viggiano, Hamza Khan, Alejandro J. Gonzalez, Geoff Thomas, Sandra Elmasry, Talha A. Malik

**Affiliations:** 1 Department of Hematology & Oncology, Mayo Clinic, Phoenix, AZ 85054, USA; jamal.fares@mayo.edu; 2 Department of Internal Medicine, Mayo Clinic, Rochester, MN 55905, USA; 3 Department of Internal Medicine, Mayo Clinic, Phoenix, AZ 85054, USAgonzalez.alejandro@mayo.edu (A.J.G.);; 4 Department of Internal Medicine, Northern Arizona Healthcare, Verde Valley, AZ 86326, USA; 5 Department of Gastroenterology & Hepatology, Mayo Clinic, Phoenix, AZ 85054, USA

**Keywords:** Crohn’s disease, perianal fistula, fistula closure, fistula remission, radiology, surgery, CRP, FCP

## Abstract

**Background**: Perianal fistulizing Crohn’s disease (CD) is associated with significant morbidity and remains difficult to treat. Although advanced medical therapies are widely used, much of the available evidence derives from heterogeneous fistula populations or luminal CD trials, with limited perianal-specific synthesis and inconsistent outcome definitions. We conducted a systematic review focusing exclusively on perianal-specific clinical, radiologic, and composite outcomes in adults with perianal fistula (PAF) CD. **Methods**: We performed a systematic review in accordance with PRISMA 2020. Electronic databases were searched from inception through November 2025. We included randomized controlled trials and cohort studies enrolling adults with CD reporting outcomes specific to PAF. Interventions included biologics and small-molecule therapies, compared with placebo or other therapies. Due to substantial heterogeneity in outcome definitions and study designs, a meta-analysis was not performed. Risk of bias was assessed using Risk of Bias 2 (RoB 2) for randomized trials and the Newcastle–Ottawa Scale for observational studies. **Results**: Seven studies including >1200 participants with PAF-CD met inclusion criteria. Follow-up ranged from 24 weeks to 5 years. Across studies, outcome definitions and assessment modalities varied. Upadacitinib demonstrated significantly higher clinical fistula closure compared with placebo across multiple dose regimens at 52 weeks. In observational comparisons, ustekinumab and vedolizumab were associated with higher clinical closure rates than anti-TNF therapies. However, infliximab demonstrated higher closure rates than adalimumab as a first-line treatment. The definition for radiologic remission was less consistent across studies and often did not parallel clinical outcomes. Composite clinical–radiologic remission and response were reported in a limited number of studies, with filgotinib showing higher composite outcomes in comparison to placebo in a phase 2 trial. Surgical interventions, relapse outcomes, biomarkers [C-reactive protein (CRP)/fecal calprotectin (FCP)], and patient-reported outcomes were variably reported and not consistently significant across comparisons. **Conclusions**: Evidence for advanced therapies in PAF CD remains limited by heterogeneity in endpoint definitions, subjectivity in clinical observation, inconsistent radiologic reporting, and reliance on subgroup or observational comparisons. While anti-TNF therapy remains the most established option in guidelines, emerging data suggest significant benefits with ustekinumab, vedolizumab, and JAK inhibitors in selected patients. There is a need for PAF-specific, adequately powered randomized trials using standardized composite clinical and radiologic endpoints.

## 1. Introduction

Perianal fistulizing Crohn’s disease (CD) represents one of the most challenging and morbid phenotypes of CD, affecting up to one-third of patients over the course of their disease [1]. Perianal fistulas (PAFs) are associated with significant pain and recurrent infections that significantly impair quality of life [2]. Additionally, they frequently require multidisciplinary care involving medical and surgical interventions [1,3]. Despite advances in medical therapy for luminal CD, effective and durable treatment of PAF remains difficult, and long-term outcomes are often suboptimal [4].

Biologic and small-molecule therapies have expanded the therapeutic landscape for PAF, with anti–tumor necrosis factor (anti-TNF) agents historically acting as the primary medical management [3]. More recently, integrin antagonists, interleukin inhibitors, and Janus kinase inhibitors (JAK inhibitors) have been evaluated for fistulizing disease [5]. However, evidence supporting these therapies is heterogeneous, derived from trials and observational studies that vary in design, outcome definitions, and follow-up duration [5]. Importantly, many studies assess “fistulizing Crohn’s disease” as a single entity, without clearly distinguishing PAF from fistulas involving other regions of the gastrointestinal tract (GI).

Perianal fistulizing disease differs from luminal CD not only in anatomical location but also in its underlying inflammatory and structural complexity, often involving deep tissue tracts, abscess formation, and ongoing septic complications [6]. Unlike luminal disease, where mucosal healing has become a standardized therapeutic target, consensus definitions of healing in PAF remain less clearly defined [3,7]. Consequently, treatment success in clinical practice often relies on clinical assessment rather than standardized composite endpoints [3,8]. This discrepancy further highlights the need for a focused evaluation of perianal-specific evidence.

Clinical endpoints such as fistula closure or resolution of drainage are commonly used, yet these may not reliably reflect underlying structural healing [9]. Radiologic confirmation of fistula healing using magnetic resonance imaging (MRI) or ultrasound is infrequently reported, and outcomes related to durability of response, relapse, surgical intervention, biomarkers, and patient-reported quality of life are inconsistently reported [10]. As a result, the true efficacy and comparative effectiveness of available medical therapies for PAF remain difficult to interpret.

Previous reviews of fistulizing CD often include pooled heterogeneous fistula populations, combine perianal and non-perianal outcomes, or are focused primarily on luminal disease activity [4]. For instance, Present et al. (1999) provided the cornerstone randomized evidence supporting infliximab for fistulizing CD, though outcomes were reported in a cohort that combined perianal and abdominal fistulas [11]. As a result, much of the evidence supporting current guideline recommendations for perianal disease is derived from extrapolated or subgroup analyses rather than trials designed specifically for PAF [12]. In addition, outcome definitions across perianal studies vary substantially, with inconsistent use of drainage-based, radiologic, and composite endpoints, limiting cross-study comparison and interpretation of durability of response [13]. A focused perianal-specific synthesis is therefore necessary to clarify the true efficacy of available therapies, identify gaps in outcome reporting, and inform both clinical decision-making and future trial design in PAF.

Therefore, we conducted a systematic review of medical therapies for PAF CD, focusing exclusively on perianal-specific outcomes. We aimed to synthesize available evidence on clinical remission and response, radiologic outcomes, surgical endpoints, relapses, biomarkers, and patient-reported outcomes.

This systematic review provides the following key contributions:

- A perianal-specific synthesis of medical therapies in CD, avoiding extrapolation from mixed fistula or luminal disease populations.
- A structured evaluation of clinical, radiologic, and combined endpoints, highlighting heterogeneity in outcome definitions across studies.
- Identification of major gaps in durability reporting, relapse assessment, and standardized composite healing definitions.
- Clarification of the limitations underlying current guideline recommendations, which are often based on subgroup or extrapolated data.

## 2. Materials and Methods

### 2.1. Study Design

**Figure 1:**
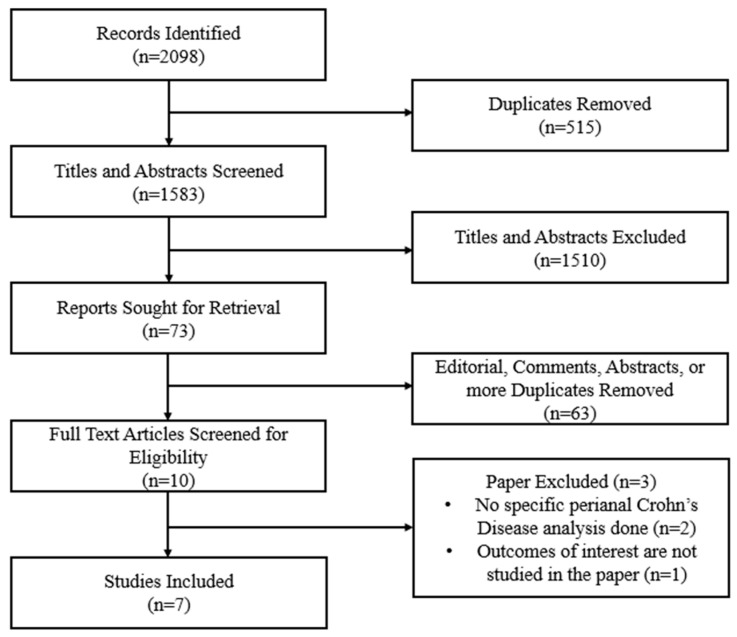
PRISMA flow diagram.

This systematic review was conducted in accordance with the Preferred Reporting Items for Systematic Reviews and Meta-Analyses (PRISMA) 2020 guidelines (Figure 1) [14]. The complete PRISMA checklist is provided in Supplementary Table S1. A review question, eligibility criteria, and outcomes of interest were defined before the start of the study. Given the substantial clinical and methodological heterogeneity across studies, including differences in outcome definitions, assessment modalities, comparators, and follow-up duration, a quantitative meta-analysis was not completed. The review protocol was prospectively registered in the International Prospective Register of Systematic Reviews (PROSPERO; Registration No. CRD420251207916).

### 2.2. Eligibility Criteria and Outcomes of Interest

We included studies involving adult patients (≥18 years) with CD and PAF, including both draining and non-draining fistulas. Studies enrolling patients with fistulas from different regions in the GI tract were eligible only if outcomes specific to PAF were reported. Studies reporting outcomes only for non-PAF or mixed PAF with other regions within the GI tract were excluded.

Eligible interventions included medical therapies used for treating PAF CD, including biologic agents and small-molecule therapies. Comparators included placebo or another medical therapy. Randomized controlled trials (RCTs) and prospective and retrospective cohort studies were eligible, whereas case reports, small case series, editorials, reviews, and conference abstracts without full text were excluded.

The primary outcomes of interest were PAF-specific clinical endpoints, including complete remission, defined as clinical closure of external PAF openings with absence of drainage, and partial remission or clinical response, defined as resolution of drainage or a reduction in draining PAF.

Secondary outcomes included radiologic PAF response or remission, PAF requiring surgery, relapse of response after PAF remission, biomarker outcomes [C-reactive protein (CRP) and fecal calprotectin (FCP)], and patient-reported quality-of-life measures. Outcomes were extracted only when reported specifically for PAF.

### 2.3. Information Sources and Study Selection

A comprehensive literature search was performed across multiple electronic databases from inception through November 2025. The search strategy combined keywords related to CD, PAF, and different medical therapies. The full search strategy is provided in Supplementary Tables S2 and S3. Reference lists of included studies and relevant reviews were manually screened to identify additional eligible studies.

After removal of duplicate records, titles and abstracts were screened independently and blindly by two reviewers (F.J. and T.S.). Full texts of potentially eligible studies were then assessed for inclusion. Disagreements at any stage of study selection were resolved by a third reviewer (H.K.). The study selection process is summarized in a PRISMA flow diagram (Figure 1).

### 2.4. Data Extraction, Risk of Bias Assessment, and Synthesis

Data were extracted independently and blindly by two reviewers (F.J. and T.S.) and disagreements were resolved by a third reviewer (T.V.). Extracted variables included study design, sample size, patient characteristics, PAF definitions, intervention and comparator details, follow-up duration, outcome definitions, and reported effect estimates. For each outcome, we recorded, when available, absolute event counts, proportions, effect measures, confidence intervals, *p*-values, and whether analyses were adjusted. Only perianal-specific outcomes were extracted.

Risk of bias was assessed independently by two reviewers (A.J.G. and G.T.). RCTs were evaluated using the Cochrane Risk of Bias 2 (Rob2) tool, and observational studies were assessed using the Newcastle–Ottawa Scale. Discrepancies were resolved by a third reviewer (F.J.) [15,16].

## 3. Results

### 3.1. Study Selection

A literature search was conducted and identified 2098 records through different databases. A total of 515 duplicate records were identified, leaving 1583 records for title and abstract screening. Deduplication was performed in two stages: 467 duplicates were removed prior to uploading records to the screening platform, and an additional 48 duplicates were identified and removed within the platform. Among these, 1510 records were excluded based on title and abstract review.

Following title and abstract screening, 73 reports were sought for retrieval. Of these, 63 were excluded prior to full-text eligibility assessment because they were editorials, comments, or conference abstracts without full published data. The remaining 10 full-text articles were assessed for eligibility. Three were excluded for not meeting inclusion criteria, resulting in seven studies included in the qualitative synthesis (Figure 1).

### 3.2. Study Characteristics

The seven included studies enrolled a total of over 1200 participants with PAF and CD, including both RCTs (n = 2) and observational cohort studies (n = 5) [17,18,19,20,21,22,23]. Follow-up duration ranged from 24 weeks to five years, reflecting differences in study design and treatment intent. The studies were conducted in different locations, mainly in North America and Europe. Five studies focused primarily on patients with PAF, while the other two studies had PAF as a subgroup analysis from a larger luminal CD group.

The use of prior anti-TNF was common in the studies, reflecting the refractory nature of many included cohorts. Concomitant immunomodulators were generally permitted, and seton placement at baseline was allowed in most trials, mirroring real-world multidisciplinary management. However, variability in baseline disease characteristics, prior biologic exposure, and outcome assessment methods limit direct cross-study comparison. Detailed study characteristics are summarized in Table 1.

### 3.3. Clinical Fistula Closure

Clinical fistula closure, defined by the absence of PAF drainage on perianal examination or documentation of remission by the treating physician, was reported in three studies [18,19,23].

One RCT, comparing upadacitinib at different dosing with placebo, demonstrated higher clinical fistula closure. The difference was statistically significant at all different doses (45 mg, 30 mg, and 15 mg), with a follow-up period of 52 weeks [18]. Notably, response appeared dose-independent, although the study was not specifically powered for perianal outcomes.

**Figure 2:**
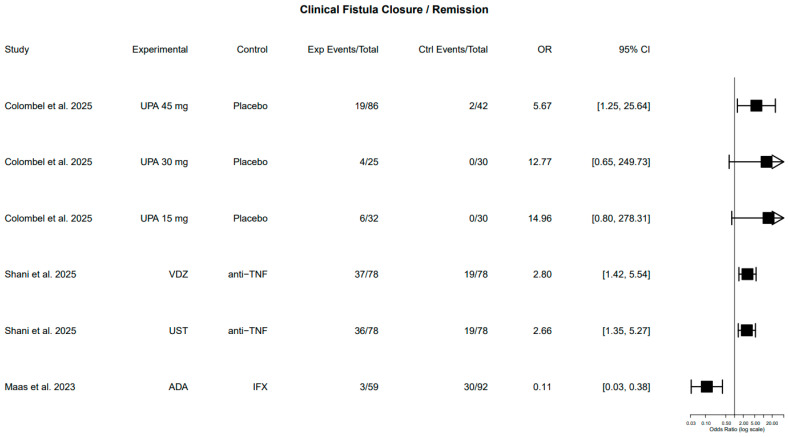
Forest plot of clinical fistula closure across included studies. CI: confidence interval, Ctrl: control, exp: experimental, OR: odds ratio [18,19,23]. Forest plot of clinical fistula closure across included studies. Clinical closure was defined as absence of perianal fistula drainage on examination or documentation of remission by the treating physician. Odds ratios and 95% confidence intervals were calculated from reported event counts (crude estimates). No pooled effect estimate was generated due to substantial clinical and methodological heterogeneity across studies, including differences in study design, follow-up duration, and comparator groups.

Regarding the observational studies, ustekinumab and vedolizumab were associated with statistically significant higher rates of fistula closure compared with anti-TNF therapy [19]. Furthermore, adalimumab was associated with lower closure rates compared to infliximab [23]. These findings suggest potential differences in effectiveness across agents; however, given the non-randomized nature of these comparisons and variability in baseline disease severity and prior biologic exposure, causal inferences remain limited. Effect estimates, *p*-values, and follow-up period are summarized in Table 2 and visually illustrated in Figure 2.

### 3.4. Clinical Fistula Response

Clinical fistula response, defined as improvement in PAF, was assessed in four studies [18,19,21,23]. As expected, response rates were higher than complete closure rates across all therapies, reflecting the more permissive definition of improvement compared with full remission.

Upadacitinib was associated with a significantly higher clinical fistula response compared to placebo at different upadacitinib doses [18]. Similarly, when infliximab was compared to placebo, infliximab demonstrated a statistically significant clinical response rate, consistent with its established role in fistulizing CD [21]. Furthermore, comparing ustekinumab with anti-TNF and infliximab with adalimumab showed significantly higher clinical response rate in the ustekinumab and infliximab cohorts, respectively [19,23]. In contrast, vedolizumab had a higher clinical response rate than infliximab, but the association was not statistically significant [19].

**Figure 3:**
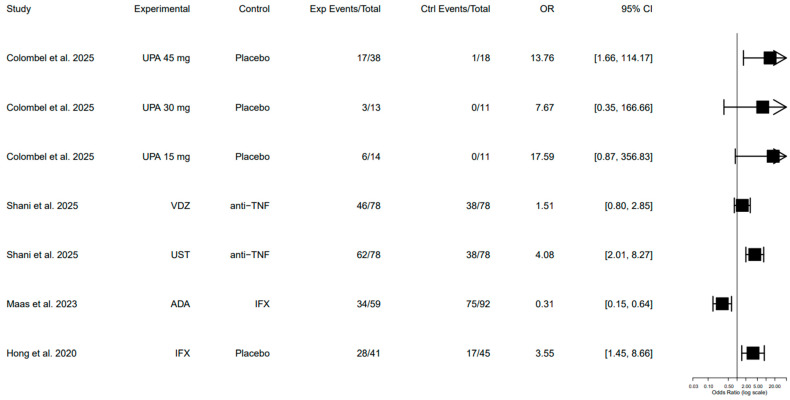
Forest plot of clinical fistula response across advanced therapies. CI: confidence interval, Ctrl: control, exp: experimental, OR: odds ratio [18,19,21,23]. Forest plot of clinical fistula response across included studies. Clinical response was defined as improvement in fistula drainage or ≥50% reduction in drainage, as reported by individual studies. Odds ratios and 95% confidence intervals were calculated from reported event counts (crude estimates). No pooled effect estimate was generated due to substantial clinical and methodological heterogeneity across studies, including differences in outcome definitions and follow-up duration.

Overall, while multiple agents were associated with clinical improvement, variability in study design, comparator arms, and baseline patient characteristics limits direct comparison of response magnitude across therapies. Table 3 summarizes the clinical fistula response across different studies and is visually illustrated in Figure 3.

### 3.5. Radiological Outcome

Radiologic remission, defined by MRI-based closure of fistula tracts and absence of abscesses, was reported in two studies [17,19]. While numerically higher rates of radiologic remission were observed with ustekinumab and vedolizumab compared with anti-TNF therapy, these differences did not consistently reach statistical significance. Radiological outcome details are summarized in Supplementary Table S4.

### 3.6. Combined Fistula Remission Outcome

Combined fistula remission outcomes were defined as radiological and clinical remission of the PAF. One RCT was included in this outcome [20]. Filgotinib, a JAK inhibitor, was associated with a higher combined remission rate compared to placebo at 200 mg and 100 mg dose. Details regarding the combined fistula remission outcomes are summarized in Supplementary Table S5.

### 3.7. Combined Fistula Response Outcome

Two studies, one RCT and one observational, documented combined fistula response outcomes [17,20]. Combined fistula response outcome was defined as improvement in PAF that was confirmed both clinically and radiologically. Filgotinib was associated with a better response compared to placebo. However, when comparing ustekinumab with infliximab, the difference was not statistically significant. Combined fistula response outcomes are summarized in Supplementary Table S6.

### 3.8. Surgical Outcome

Three observational studies reported surgical outcomes in PAF CD patients, defined as the need for surgery during the study period [17,19,22]. No statistically significant differences in surgical intervention rates were observed across treatment comparisons. Supplementary Table S7 summarizes the surgical outcomes.

### 3.9. Relapses After Remission Outcome

Studies reporting relapse PAF rates were also assessed. Relapses were defined as recurrence of symptoms or abscess in the perianal region. Three studies evaluated this outcome and all were observational [17,19,22]. Statistical significance was observed when ustekinumab was compared with infliximab, anti-TNF with placebo, and ustekinumab with placebo. Supplementary Table S8 summarizes relapse rate outcomes.

### 3.10. CRP Change Outcome

Two studies, one RCT and one observational, reported changes in CRP [17,20]. Although greater CRP reductions were observed with infliximab compared to ustekinumab, this difference was not statistically significant [17]. Furthermore, filgotinib showed a higher CRP decrease compared to placebo [20]. Details regarding CRP changes are summarized in Supplementary Table S9.

### 3.11. FCP Change Outcome

One RCT reported FCP changes between filgotinib and placebo [20]. Filgotinib at different doses demonstrated a decrease in FCP while placebo showed an increase in FCP during the study period. Supplementary Table S10 summarizes FCP outcomes.

### 3.12. Subjective Outcome

Subjective outcomes of PAF in CD were also extracted [20,21]. Different scoring systems were used in different studies. When compared to placebo, filgotinib and infliximab showed a more positive subjective response. Details regarding the subjective outcomes are summarized in Supplementary Table S11.

### 3.13. Risk of Bias

Overall risk of bias was rated as low to moderate across the included studies. RCTs were judged to have “some concerns” overall, with concerns primarily related to deviations from intended interventions, missing outcome data, outcome measurement in certain domains, and selection of reported result. Observational studies were limited by residual confounding and heterogeneous outcome definitions. A detailed assessment of study quality is provided in Table 4 and Table 5.

## 4. Discussion

In this systematic review, we synthesized evidence from seven studies including over 1200 patients with PAF CD treated with advanced medical therapies. Overall, rates of complete fistula closure and response varied substantially across therapies and outcome definitions. Among randomized data, upadacitinib demonstrated significantly higher rates of clinical fistula closure compared with placebo across multiple dosing regimens, while filgotinib was associated with higher combined clinical–radiologic remission and response compared with placebo. In observational cohorts, ustekinumab and vedolizumab were consistently associated with higher clinical fistula closure and response rates compared with anti-TNF therapy, while infliximab showed higher closure and response rates than adalimumab. Radiological remission rates were numerically higher with ustekinumab and vedolizumab but did not consistently reach statistical significance. Surgical outcomes and relapse rates varied across studies, with some evidence suggesting lower relapse rates with ustekinumab compared with infliximab. Together, these findings highlight meaningful heterogeneity in therapeutic response and underscore the lack of a single clearly superior medical strategy for PAF.

Management of PAF CD remains challenging and different from luminal CD. Current American Gastroenterological Association (AGA) and American College of Gastroenterology (ACG) guidelines strongly recommend infliximab for fistulizing CD based largely on RCT data, which showed fistula closure and response, with adalimumab supported by less strong evidence [3,4]. However, this recommendation is mainly based on the Present et al. RCT, which combined abdominal and PAF [11]. Ustekinumab and vedolizumab are suggested as alternatives, but these recommendations are mainly extrapolated from luminal CD trials rather than studies specifically designed for PAF [3,4]. Guidelines often group all fistulizing disease together, despite the biological and clinical differences between luminal, enterocutaneous, and PAF.

Beyond clinical heterogeneity, perianal fistulizing CD is biologically distinct from luminal inflammation. Fistula formation reflects a complex interplay between epithelial–to-mesenchymal transition, persistent transmural inflammation, local sepsis, and aberrant wound healing pathways, including overexpression of transforming growth factor–β and matrix metalloproteinases [24]. Unlike luminal CD, where mucosal healing has emerged as a validated therapeutic target, structural fistula healing requires resolution of deep tissue tracts and associated abscess cavities, processes that may not parallel mucosal response [25]. This biological divergence likely explains why therapies demonstrating robust luminal efficacy do not uniformly translate into durable fistula closure. It also highlights why reliance on luminal CD trial data may overestimate effectiveness in perianal disease.

The need for standardized composite endpoints in PAF has been increasingly emphasized [26]. Radiologic assessment using pelvic MRI has demonstrated that clinical closure does not consistently correlate with true tract healing, and persistent subclinical inflammation may predict relapse despite apparent clinical remission [9]. Recent consensus statements advocate incorporation of objective radiologic measures alongside drainage-based assessments to define deep remission in perianal disease [7]. Without such standardized definitions, variability in endpoint reporting continues to limit cross-study comparison and therapeutic sequencing decisions. A treat-to-target framework specifically adapted for perianal disease, incorporating both structural and symptomatic endpoints, may provide a more rational strategy for evaluating emerging biologic and small-molecule therapies.

Our findings directly address this critical evidence gap in the management of perianal CD. While anti-TNF remains the guideline-recommended first-line treatment, observational data from this review suggest that ustekinumab and vedolizumab may achieve comparable or superior clinical fistula outcomes in selected patients, particularly those with prior anti-TNF exposure. In parallel, emerging small-molecule data from JAK inhibition (upadacitinib and filgotinib) demonstrate promising results for fistula closure and combined remission. However, these findings are largely derived from subgroup or early-phase analyses and require confirmation in trials specifically powered by PAF.

These results support a personalized, multidisciplinary approach to PAF management. Medical therapy alone is unlikely to be sufficient in many patients, and most included studies allowed concomitant immunomodulators and baseline seton placement, reflecting real-world practice. The observed variability in outcomes across therapies also emphasizes the importance of clearly defining treatment goals, whether clinical closure, radiologic healing, or combined remission. Radiologic endpoints, while increasingly used, did not consistently align with clinical outcomes, reinforcing the need for standardized composite definitions in future trials.

Importantly, much of the evidence informing current clinical practice is derived from heterogeneous or secondary analyses rather than trials designed specifically for perianal fistulizing disease [10]. Even landmark randomized trials frequently combined perianal and non-perianal fistulas or relied on drainage-based endpoints without systematic radiologic confirmation [12]. Observational comparisons, while clinically informative, remain vulnerable to confounding by indication, particularly in patients with prior biologic exposure or complex fistula anatomy [27]. Furthermore, inconsistent definitions of remission and response limit cross-trial comparison and likely contribute to the wide variability in reported efficacy. Without standardized composite endpoints incorporating both clinical and radiologic assessment, interpretation of durability and true structural healing remain incomplete. Future studies should prioritize perianal-specific primary endpoints, incorporate standardized radiologic assessment alongside clinical evaluation, and report long-term durability outcomes. Dedicated randomized trials focused exclusively on PAF are needed to reduce reliance on extrapolated or subgroup data and to better inform therapeutic sequencing strategies.

These findings also carry broader conceptual implications. The current literature reflects a paradigm in which perianal fistulizing disease is often treated as an extension of luminal CD rather than a biologically and structurally distinct phenotype. Reframing PAF as a separate therapeutic entity may require redefining treatment targets toward long-term structural healing rather than short-term drainage control. Establishing a unified conceptual framework for remission in PAF could improve consistency in trial design and ultimately refine treatment algorithms.

This review demonstrates several key strengths. It focuses specifically on PAF CD rather than extrapolating from luminal disease, systematically extracted multiple clinically relevant outcomes, and integrated evidence from both randomized and real-world data. However, several important limitations should be acknowledged. Substantial heterogeneity in outcome definitions, follow-up duration, and comparator arms precluded quantitative meta-analysis. Many observational studies were subject to confounding by indication, with inconsistent reporting of adjusted effect estimates. Additionally, several outcomes were derived from subgroup or post hoc analyses rather than trials designed primarily for PAF, thereby limiting internal validity.

## 5. Conclusions

In this systematic review of advanced medical therapies for perianal fistulizing CD, the available evidence demonstrates heterogeneous efficacy across clinical, radiologic, and composite endpoints. Although anti-TNF therapy remains the most established treatment based on randomized data, much of the perianal-specific evidence for alternative biologic and small-molecule agents derives from subgroup analyses or observational comparisons with methodological limitations. Variability in outcome definitions, inconsistent incorporation of radiologic assessment, and differences in prior biologic exposure restrict direct cross-study comparisons and limit definitive conclusions regarding comparative effectiveness. These findings highlight the need for powered perianal-specific randomized trials using standardized composite clinical and radiologic endpoints to better inform guideline development and clinical decision-making.

## Figures and Tables

**Table 1:** Characteristics of studies evaluating advanced medical therapies for perianal Crohn’s disease.

Author (Year)	Country	Study Design	Population (Perianal CD)	N (Total)	Therapy (Experimental vs. Control)	Prior Anti-TNF Allowed	Concomitant Immunomodulator	Seton at Baseline	Follow-Up
Chen et al. (2025) [17]	China	Cohort	Specific	97	UST vs. IFX	No	Yes	Yes	For UST: 10.1 mo For IFX: 31 mo
Colombel et al. (2025) [18]	USA	RCT	Subgroup	244	UPA vs. Placebo	Yes	Yes	Yes	52 wks
Shani et al. (2025) [19]	Europe and Canada	Cohort	Specific	234	VDZ vs. anti-TNF AND UST vs. anti-TNF	Yes	Yes	Yes	6 mo
Reinisch et al. (2024) [20]	Austria, USA, Spain, Netherlands, Spain, Belgium	RCT	Specific	57	filgotinib vs. placebo	Yes	Yes	No	24 wks
Gubatan et al. (2023) [22]	USA	Cohort	Specific	311	Anti-TNF vs. placebo AND VDZ vs. placebo AND UST vs. placebo	Yes	Yes	Yes	5 yrs
Maas et al. (2023) [23]	USA	Cohort	Specific	151	ADA vs. IFX	No	Yes	Yes	12 mo
Hong et al. (2020) [21]	Shanghai and China	Cohort	Specific	86	IFX vs. placebo	No	NR	Yes	6–12 mo

ADA, adalimumab; Anti-TNF: anti-tumor necrosis factor; CD, Crohn’s disease; IFX, infliximab; NR, not reported; RCT, randomized controlled trial; USA: United States of America, UPA, upadacitinib; UST, ustekinumab; VDZ, vedolizumab. In Shani et al., the anti-TNF comparator consisted of infliximab and adalimumab. In Gubatan et al., anti-TNF therapy included infliximab, adalimumab, and certolizumab (pooled analysis).

**Table 2:** Clinical fistula closure rates across advanced therapies for perianal Crohn’s disease.

Author (Year)	Design	Therapy (Exp vs. Ctrl)	Definition Used	Follow-Up	Exp n/N (%)	Ctrl n/N (%)	Effect Measure	*p*-Value
Colombel et al., (2025) [18]	RCT	45 mg UPA vs. Placebo	Closure of external perianal fistula openings	52 wks	19/86 (22.1)	2/42 (4.8)	NR	0.013
Colombel et al., (2025) [18]	RCT	30 mg UPA vs. Placebo	Closure of external perianal fistula openings	52 wks	4/25 (16)	0/30	NR	0.037
Colombel et al., (2025) [18]	RCT	15 mg UPA vs. Placebo	Closure of external perianal fistula openings	52 wks	6/32 (18.8)	0/30	NR	0.024
Shani et al., (2025) [19]	Cohort	VDZ vs. anti-TNF	Complete resolution of signs and symptoms associated with perianal fistulas.	6 mo	37/78 (47.4)	19/78 (24)	aOR	0.02
Shani et al., (2025) [19]	Cohort	UST vs. anti-TNF	Complete resolution of signs and symptoms associated with perianal fistulas.	6 mo	36/78 (46.1)	19/78 (24)	aOR	0.007
Maas et al., (2023) [23]	Cohort	ADA vs. IFX	Fistula was without drainage and closed on perianal exam and/or the clinician documented in their assessment that perianal disease was in remission.	12 mo	32.7%	5.3%	aOR	0.043

ADA: adalimumab, anti-TNF: anti–tumor necrosis factor, aOR: adjusted odds ratio, ctrl: control, exp: experimental, IFX: infliximab, mo: months, NR: not reported, RCT: randomized controlled trial, UPA: upadacitinib, UST: ustekinumab, VDZ: vedolizumab, wks weeks. In Shani et al., the anti-TNF comparator consisted of infliximab and adalimumab (pooled analysis). In Gubatan et al., anti-TNF therapy included infliximab, adalimumab, and certolizumab (pooled analysis).

**Table 3:** Clinical fistula response rates across advanced therapies for perianal Crohn’s disease.

Author (Year)	Design	Therapy (Exp vs. Ctrl)	Definition Used	Follow-Up	Exp n/N (%)	Ctrl n/N (%)	Effect Measure	*p*-Value
Colombel et al., (2025) [18]	RCT	45 mg UPA vs. Placebo	Healed fistulas with scars were considered resolved	52 wks	17/38 (44.7)	1/18 (5.6)	NR	0.003
Colombel et al., (2025) [18]	RCT	30 mg UPA vs. Placebo	Healed fistulas with scars were considered resolved	52 wks	3/13 (23.1)	0/11	NR	0.105
Colombel et al., (2025) [18]	RCT	15 mg UPA vs. Placebo	Healed fistulas with scars were considered resolved	52 wks	6/14 (28.6)	0/11	NR	0.223
Shani et al., (2025) [19]	Cohort	VDZ vs. anti-TNF	Any degree of improvement in perianal disease, including clinical perianal remission, as determined by the treating physician during clinic visits at least 6 months after treatment initiation.	6 mo	46/78 (59)	38/78 (48.7)	aOR	0.453
Shani et al., (2025) [19]	Cohort	UST vs. anti-TNF	Any degree of improvement in perianal disease, including clinical perianal remission, as determined by the treating physician during clinic visits at least 6 months after treatment initiation.	6 mo	62/78 (79.4)	38/78 (48.7)	aOR	<0.001
Maas et al., (2023) [23]	Cohort	ADA vs. IFX	50% decrease in fistula drainage noted on perianal exam with application of gentle pressure at the fistula opening(s) compared to baseline exam and/or clinician assessment noted fistula improvement	12 mo	82%	57.9%	aOR	0.01
Hong et al., (2020) [21]	Cohort	IFX vs. placebo	≥50% reduction from baseline in the number of draining Perianal fistulas, as determined by gentle compression.	6–12 mo	28/41 (68.3)	17/45 (37.8)	NR	0.005

ADA: adalimumab, anti-TNF: anti–tumor necrosis factor, aOR: adjusted odds ratio, ctrl: control, exp: experimental, IFX: infliximab, mo: months, NR: not reported, RCT: randomized controlled trial, UPA: upadacitinib, UST: ustekinumab, VDZ: vedolizumab, wks weeks. In Shani et al., the anti-TNF comparator consisted of infliximab and adalimumab (pooled analysis).

**Table 4:** Risk of bias assessment for randomized controlled trials using Cochrane Risk of Bias 2 tool.

Author (Year)	Randomization Process	Deviations from Intended Interventions	Missing Outcome Data	Measurement of Outcome	Selection of Reported Result	Overall
Colombel et al. (2025) [18]	Some	Some	Low	Some	Some	Some
Reinisch et al. (2024) [20]	Low	Low	Some	Low	Low	Some

**Table 5:** Risk of bias assessment for observational cohort studies (Newcastle–Ottawa scale).

Author (Year)	Representativeness of Exposed Cohort	Selection of Non-Exposed Cohort	Ascertainment of Exposure	Outcome Absent at Baseline	Control for Main Confounders	Outcome Assessment	Follow-Up Adequacy	Completeness of Follow-Up	NOS Score (/9)
Chen et al. (2025) [17]	Limited	Same center/time	Secure records	Yes	Yes	MRI + clinical	Adequate	Incomplete	7/9
Shani et al. (2025) [19]	Good	Same cohort	Secure records	Yes	Yes	Physician-based	Adequate	Incomplete	7/9
Gubatan et al. (2023) [22]	Limited	Same cohort	Secure records	Yes	Yes	Standardized clinical tools	Long	Incomplete	7/9
Maas et al. (2023) [23]	Good	Same cohort	Secure records	Yes	Yes	Clinical exam	Adequate	Complete	8/9
Hong et al. (2020) [21]	Limited	Same cohort	Secure records	Yes	Partial	Validated tools	Adequate	Near complete	7/9

## Data Availability

No new data were created or analyzed in this study.

## Supplementary Materials

The following supporting information can be downloaded at: https://www.mdpi.com/article/10.3390/ph19030417/s1, Table S1: PRISMA checklist. Table S2: Search Strategy Used for Study Identification. Table S3: Database Yield and Deduplication. Table S4: Radiological Fistula Remission Rates Across Advanced Therapies for Perianal Crohn’s Disease. Table S5: Combined Fistula Remission Rates (Radiological and Clinical) Across Advanced Therapies for Perianal Crohn’s Disease. Table S6: Combined Fistula Response Rates (Radiological and Clinical) Across Advanced Therapies for Perianal Crohn’s Disease. Table S7: Surgery Outcomes Across Advanced Therapies for Perianal Crohn’s Disease. Table S8: Relapse Rates Across Advanced Therapies for Perianal Crohn’s Disease. Table S9: CRP Change Across Advanced Therapies for Perianal Crohn’s Disease. Table S10: FCP Change Across Advanced Therapies for Perianal Crohn’s Disease. Table S11: Subjective Outcome Across Advanced Therapies for Perianal Crohn’s Disease.
