# Supplementary material for: Advanced Medical Therapies for Perianal Fistulizing Crohn’s Disease: A Systematic Review of Clinical, Radiological, Surgical, and Composite Outcomes
Source: Pharmaceuticals (Basel). 2026 Mar 4;19(3):417. doi: 10.3390/ph19030417 (PMC13029034; doi:10.3390/ph19030417)
Supplement: Supplementary file 1 [file pharmaceuticals-19-00417-s001.zip › Tables S2-S11.pdf]

Supplementary Table S2: Search Strategy Used for Study Identification.

Electronic Databases	Search	Search Strategy	Results
Embase	1	(perianal adj1 crohn*).mp. [mp=title, abstract, heading word, drug trade name, original title, device manufacturer, drug manufacturer, device trade name, keyword heading word, floating subheading word, candidate term word]	1,512
	2	(refractory adj1 crohn*).mp. [mp=title, abstract, heading word, drug trade name, original title, device manufacturer, drug manufacturer, device trade name, keyword heading word, floating subheading word, candidate term word]	875
	3	exp Crohn disease/dt [Drug Therapy]	25,571
	4	crohn*.mp.	147,549
	5	(((((perianal or anal or anorect* or anal) adj1 fistula) or fistula or fistulas or fistulizing or perianal) adj1 fistulizing) or abscess or abscesses).mp. [mp=title, abstract, heading word, drug trade name, original title, device manufacturer, drug manufacturer, device trade name, keyword heading word, floating subheading word, candidate term word]	173,828
	6	3 or 4	147,549
	7	5 and 6	9,012
	8	1 or 2	2,372
	9	exp Janus kinase inhibitor/	47,594
	10	exp antirheumatic agent/	1,334,74 7
	11	exp biosimilar agent/	9,549
	12	(monoclonal adj1 antibod*).mp. [mp=title, abstract, heading word, drug trade name, original title, device manufacturer, drug manufacturer, device trade name, keyword heading word, floating subheading word, candidate term word]	397,510
	13	(tumor adj1 necrosis adj1 factor adj1 inhibitor*).mp. [mp=title, abstract, heading word, drug trade name, original title, device manufacturer, drug manufacturer, device trade name, keyword heading word, floating subheading word, candidate term word]	33,161
	14	(tnf adj1 inhibitor*).mp. [mp=title, abstract, heading word, drug trade name, original title, device manufacturer, drug manufacturer, device trade name, keyword heading word, floating subheading word, candidate term word]	7,857
	15	mirikizumab.mp. [mp=title, abstract, heading word, drug trade name, original title, device manufacturer, drug manufacturer, device trade name, keyword heading word, floating subheading word, candidate term word]	808
	16	Adalimumab.mp. [mp=title, abstract, heading word, drug trade name, original title, device manufacturer, drug manufacturer, device trade name, keyword heading word, floating subheading word, candidate term word]	55,743
	17	Certolizumab.mp. [mp=title, abstract, heading word, drug trade name, original title, device manufacturer, drug manufacturer, device trade name, keyword heading word, floating subheading word, candidate term word]	11,858
	18	golimumab.mp. [mp=title, abstract, heading word, drug trade name, original title, device manufacturer, drug manufacturer, device trade name, keyword heading word, floating subheading word, candidate term word]	12,503
	19	vedolizumab.mp. [mp=title, abstract, heading word, drug trade name, original title, device manufacturer, drug manufacturer, device trade name, keyword heading word, floating subheading word, candidate term word]	12,005
	20	Ustekinumab.mp. [mp=title, abstract, heading word, drug trade name, original title, device manufacturer, drug manufacturer, device trade name, keyword heading word, floating subheading word, candidate term word]	17,854

	21	risankizumab.mp. [mp=title, abstract, heading word, drug trade name, original title, device manufacturer, drug manufacturer, device trade name, keyword heading word, floating subheading word, candidate term word]	3,241
	22	guselkumab.mp. [mp=title, abstract, heading word, drug trade name, original title, device manufacturer, drug manufacturer, device trade name, keyword heading word, floating subheading word, candidate term word]	3,923
	23	Natalizumab.mp. [mp=title, abstract, heading word, drug trade name, original title, device manufacturer, drug manufacturer, device trade name, keyword heading word, floating subheading word, candidate term word]	16,039
	24	tofacitinib.mp. [mp=title, abstract, heading word, drug trade name, original title, device manufacturer, drug manufacturer, device trade name, keyword heading word, floating subheading word, candidate term word]	14,783
	25	upadacitinib.mp. [mp=title, abstract, heading word, drug trade name, original title, device manufacturer, drug manufacturer, device trade name, keyword heading word, floating subheading word, candidate term word]	5,547
	26	GLPG0634.mp. [mp=title, abstract, heading word, drug trade name, original title, device manufacturer, drug manufacturer, device trade name, keyword heading word, floating subheading word, candidate term word]	92
	27	ozanimod.mp. [mp=title, abstract, heading word, drug trade name, original title, device manufacturer, drug manufacturer, device trade name, keyword heading word, floating subheading word, candidate term word]	1,740
	28	etrasimod.mp. [mp=title, abstract, heading word, drug trade name, original title, device manufacturer, drug manufacturer, device trade name, keyword heading word, floating subheading word, candidate term word]	534
	29	anti-tnf.mp. [mp=title, abstract, heading word, drug trade name, original title, device manufacturer, drug manufacturer, device trade name, keyword heading word, floating subheading word, candidate term word]	31,373
	30	anti-integrin.mp. [mp=title, abstract, heading word, drug trade name, original title, device manufacturer, drug manufacturer, device trade name, keyword heading word, floating subheading word, candidate term word]	1,591
	31	antiIL23.mp. [mp=title, abstract, heading word, drug trade name, original title, device manufacturer, drug manufacturer, device trade name, keyword heading word, floating subheading word, candidate term word]	1
	32	anti-IL23.mp. [mp=title, abstract, heading word, drug trade name, original title, device manufacturer, drug manufacturer, device trade name, keyword heading word, floating subheading word, candidate term word]	191
	33	(jak adj1 inhibitor*).mp. [mp=title, abstract, heading word, drug trade name, original title, device manufacturer, drug manufacturer, device trade name, keyword heading word, floating subheading word, candidate term word]	10,444
	34	(janus adj1 kinase adj1 inhibitor*).mp. [mp=title, abstract, heading word, drug trade name, original title, device manufacturer, drug manufacturer, device trade name, keyword heading word, floating subheading word, candidate term word]	13,485
	35	(((s1p adj1 modulator*) or sphingosine-1-phosphate) adj1 modulator*).mp. [mp=title, abstract, heading word, drug trade name, original title, device manufacturer, drug manufacturer, device trade name, keyword heading word, floating subheading word, candidate term word]	243
	36	9 or 10 or 11 or 12 or 13 or 14 or 15 or 16 or 17 or 18 or 19 or 20 or 21 or 22 or 23 or 24 or 25 or 26 or 27 or 28 or 29 or 30 or 31 or 32 or 33 or 34 or 35	1,710,030
	37	1 or 2 or 3 or 4	147,549
	38	5 and 36 and 37	3,844
	39	limit 38 to english language	3,680
	40	limit 39 to (books or chapter or conference abstract or conference paper or "conference review" or data paper or editorial or erratum or letter or note or "review" or short survey or tombstone)	2,064

	41	39 not 40	1,616
	42	limit 39 to (clinical trial or randomized controlled trial or controlled clinical trial or multicenter study or phase 1 clinical trial or phase 2 clinical trial or phase 3 clinical trial or phase 4 clinical trial)	733
**Cochrane**	1	crohn*.mp. [mp=title, original title, abstract, floating sub-heading word, mesh headings, heading words, keyword]	6,654
	2	("Janus Kinase Inhibitor*" or "Antirheumatic Agent*" or "Biosimilar Pharmaceutical*" or mirikizumab or "monoclonal antibod*" or "Tumor Necrosis Factor Inhibitor*" or "TNF-α inhibitor*" or "Biological Product*").mp. [mp=title, original title, abstract, floating sub-heading word, mesh headings, heading words, keyword]	20,703
	3	(infliximab or Adalimumab or Certolizumab or golimumab or vedolizumab or Ustekinumab or risankizumab or guselkumab or Natalizumab or tofacitinib or upadacitinib or GLPG0634 or ozanimod or etrasimod or anti-tnf or anti-integrin or anti-IL123 or anti-il23).mp. [mp=title, original title, abstract, floating sub-heading word, mesh headings, heading words, keyword]	13,418
	4	("jak inhibitor*" or "s1p modulator*" or "sphingosine-1-phosphate modulator*").mp. [mp=title, original title, abstract, floating sub-heading word, mesh headings, heading words, keyword]	1,000
	5	2 or 3 or 4	30,749
	6	(perianal or anal or anorect* or "anal fistula" or fistulas or fistulizing or "perianal fistulizing" or abscess or abscesses).mp. [mp=title, original title, abstract, floating sub-heading word, mesh headings, heading words, keyword]	14,062
	7	1 and 5	2,675
	8	6 and 7	308
	9	(rct.ti,ab. or randomized.mp.) adj1 control*.mp. adj1 trial*.mp. [mp=title, original title, abstract, floating sub-heading word, mesh headings, heading words, keyword]	736,702
	10	8 and 9	136
	11	(conference* or registry).mp. [mp=title, original title, abstract, floating sub-heading word, mesh headings, heading words, keyword]	195,924
	12	10 not 11	77
	13	limit 12 to english language	75
**EBM Reviews**	1	crohn*.mp. [mp=title, original title, abstract, floating sub-heading word, mesh headings, heading words, keyword]	6,654
	2	("Janus Kinase Inhibitor*" or "Antirheumatic Agent*" or "Biosimilar Pharmaceutical*" or mirikizumab or "monoclonal antibod*" or "Tumor Necrosis Factor Inhibitor*" or "TNF-α inhibitor*" or "Biological Product*").mp. [mp=title, original title, abstract, floating sub-heading word, mesh headings, heading words, keyword]	20,703
	3	(infliximab or Adalimumab or Certolizumab or golimumab or vedolizumab or Ustekinumab or risankizumab or guselkumab or Natalizumab or tofacitinib or upadacitinib or GLPG0634 or ozanimod or etrasimod or anti-tnf or anti-integrin or anti-IL123 or anti-il23).mp. [mp=title, original title, abstract, floating sub-heading word, mesh headings, heading words, keyword]	13,418
	4	("jak inhibitor*" or "s1p modulator*" or "sphingosine-1-phosphate modulator*").mp. [mp=title, original title, abstract, floating sub-heading word, mesh headings, heading words, keyword]	1,000
	5	2 or 3 or 4	30,749
	6	(perianal or anal or anorect* or "anal fistula" or fistulas or fistulizing or "perianal fistulizing" or abscess or abscesses).mp. [mp=title, original title, abstract, floating sub-heading word, mesh headings, heading words, keyword]	14,062
	7	1 and 5	2,675

	8	6 and 7	308
	9	(rct.ti,ab. or randomized.mp.) adj1 control*.mp. adj1 trial*.mp. [mp=title, original title, abstract, floating sub-heading word, mesh headings, heading words, keyword]	736,702
	10	8 and 9	136
	11	(conference* or registry).mp. [mp=title, original title, abstract, floating sub-heading word, mesh headings, heading words, keyword]	195,924
	12	10 not 11	77
	13	limit 12 to english language	75
PubMed	1	) ((((("perianal Crohn*") OR ("refractory crohn*")) OR ((("Crohn Disease"[Mesh]) OR (crohn*)) AND (perianal OR anal OR anorect* OR "anal fistula" OR fistula OR fistulas OR fistulizing OR "perianal fistulizing" OR abscess OR abscesses))) AND ((((((((((((((((((((((((((("Janus Kinase Inhibitors" [Pharmacological Action]) OR "Antirheumatic Agents" [Pharmacological Action]) OR ("Biosimilar Pharmaceuticals"[Mesh])) OR (mirikizumab)) OR ("Antibodies, Monoclonal, Humanized"[Mesh])) OR ("Tumor Necrosis Factor Inhibitors"[Mesh])) OR ("TNF-α inhibitor*")) OR ("Biological Products"[Mesh])) OR ("Antibodies, Monoclonal"[Mesh])) OR ("Infliximab"[Mesh])) OR ("Adalimumab"[Mesh])) OR ("Certolizumab Pegol"[Mesh])) OR ("golimumab" [Supplementary Concept])) OR ("vedolizumab" [Supplementary Concept])) OR ("Ustekinumab"[Mesh])) OR ("risankizumab" [Supplementary Concept])) OR ("guselkumab" [Supplementary Concept])) OR ("Natalizumab"[Mesh])) OR ("tofacitinib" [Supplementary Concept])) OR ("upadacitinib" [Supplementary Concept])) OR ("GLPG0634" [Supplementary Concept])) OR ("ozanimod" [Supplementary Concept])) OR ("etrasimod" [Supplementary Concept])) OR (anti-tnf)) OR (anti-integrin)) OR (anti-IL12/23)) OR (anti-il23)) OR ("jak inhibitor*")) OR ("janus kinase inhibitor*")) OR ("s1p modulator*")) OR ("sphingosine-1-phosphate modulator*")))) AND ((((("Randomized Controlled Trial" [Publication Type]) OR "Clinical Trial" [Publication Type]) OR "Retrospective Studies"[Mesh]) OR "Prospective Studies"[Mesh]) OR "Cohort Studies"[Mesh]) Filters: English	604

Supplementary Table S3: Database Yield and Deduplication.

Database	Records Identified	Duplicates Removed	Records After Deduplication
PubMed	604	3	601
Embase	733	94	639
Scopus	430	200	230
Web of Science	253	129	124
Cochrane CENTRAL	75	40	35
Author-supplied	3	1	2
**Total**	**2,098**	**467**	**1,631**

Supplementary Table S4: Radiological Fistula Remission Rates Across Advanced Therapies for Perianal Crohn's Disease.

Author (year)	Design	Therapy (Exp vs Ctrl)	Definition Used	Follow-up	Exp n/N (%)	Ctrl n/N (%)	Effect Measure	p-value
Chen et al., (2025)	Cohort	UST vs IFX	closure of all draining fistulas present at baseline and no fluid collections of > 1 cm in at least 2 dimensions on pelvic MRI	For UST: 10.1 mo<br>For IFX: 31 mo	8/49 (16.3)	11/48 (22.9)	NR	0.419
Shani et al., (2025)	Cohort	VDZ vs anti-TNF	the complete closure of perianal fistulas and the total absence of any inflammatory signs or perianal abscess, as detected by pelvic MRI or TRUS.	6 mo	12/46 (26.1)	14/58 (24.1)	aOR	0.782
Shani et al., (2025)	Cohort	UST vs anti-TNF	the complete closure of perianal fistulas and the total absence of any inflammatory signs or perianal abscess, as detected by pelvic MRI or TRUS.	6 mo	19/49 (38.8)	14/58 (24.1)	aOR	0.128

anti-TNF: anti–tumor necrosis factor, aOR: adjusted odds ratio, ctrl: control, exp: experimental, IFX: infliximab, mo: months, NR: not reported, RCT: randomized controlled trial, UPA: upadacitinib, UST: ustekinumab, VDZ: vedolizumab

In Shani et al., the anti-TNF comparator consisted of infliximab and adalimumab (pooled analysis).

Supplementary Table S5: Combined Fistula Remission Rates (Radiological and Clinical) Across Advanced Therapies for Perianal Crohn's Disease.

Author (year)	Design	Therapy (Exp vs Ctrl)	Definition Used	Follow-up	Exp n/N (%)	Ctrl n/N (%)	Effect Measure	p-value
Reinisch et al.., (2024)	RCT	200 mg filgotinib vs placebo	closure of all draining EOs present at baseline, as determined by physical assessment, and no fluid collections of >1 cm in at least two dimensions on pelvic MRI, at Week 24	24 wks	8/17 (47.1)	2/12 (16.7)	NR	NR
Reinisch et al.., (2024)	RCT	100 mg filgotinib vs placebo	closure of all draining EOs present at baseline, as determined by physical assessment, and no fluid collections of >1 cm in at least two dimensions on pelvic MRI, at Week 24	24 wks	6/24 (25)	2/12 (16.7)	NR	NR

ctrl: control, exp: experimental, NR: not reported, RCT: randomized controlled trial

Supplementary Table S6: Combined Fistula Response Rates (Radiological and Clinical) Across Advanced Therapies for Perianal Crohn's Disease.

Author (year)	Design	Therapy (Exp vs Ctrl)	Definition Used	Follow-up	Exp n/N (%)	Ctrl n/N (%)	Effect Measure	p-value
Chen et al., (2025)	Cohort	UST vs IFX	reduction from baseline of 50% or more in the number of draining tracts after UST or IFX therapy and no fluid collections of >1 cm in at least 2 dimensions on pelvic MRI.	For UST: 10.1 mo<br>For IFX: 31 mo	24/49 (49.0)	23/48 (47.9)	NR	0.92
Reinisch et al.., (2024)	RCT	200 mg filgotinib vs placebo	a reduction of at least one from baseline in the number of draining EOs, as determined by physical assessment, and no fluid collections of >1 cm in at least two dimensions on pelvic MRI, at Week 24	24 wks	8/17 (47.1)	3/12 (25)	NR	NR
Reinisch et al.., (2024)	RCT	100 mg filgotinib vs placebo	a reduction of at least one from baseline in the number of draining EOs, as determined by physical assessment, and no fluid collections of >1 cm in at least two dimensions on pelvic MRI, at Week 24	24 wks	7/24 (29.2)	3/12 (25)	NR	NR

ctrl: control, exp: experimental, IFX: infliximab, mo: months, NR: not reported, RCT: randomized controlled trial, UST: ustekinumab

Supplementary Table S7: Surgery Outcomes Across Advanced Therapies for Perianal Crohn's Disease.

Author (year)	Design	Therapy (Exp vs Ctrl)	Definition Used	Follow-up	Exp n/N (%)	Ctrl n/N (%)	Effect Measure	p-value
Chen et al., (2025)	Cohort	UST vs IFX	Surgery after biologic initiation	For UST: 10.1 mo<br>For IFX: 31 mo	4/49 (8.2)	6/48 (12.5)	NR	0.489
Shani et al., (2025)	Cohort	VDZ vs anti-TNF	performance of perianal surgery during the same period.	6 mo	26/78 (33.3)	27/78 (34.6)	NR	0.122
Shani et al., (2025)	Cohort	UST vs anti-TNF	performance of perianal surgery during the same period.	6 mo	16/78 (20.5)	27/78 (34.6)	NR	0.122
Gubatan et al., (2023)	Cohort	Anti-TNF vs placebo	confirmed surgery with available operative or procedure notes.	5 yrs	NR	NR	HR	0.28
Gubatan et al., (2023)	Cohort	VDZ vs placebo	confirmed surgery with available operative or procedure notes.	5 yrs	NR	NR	HR	0.78
Gubatan et al., (2023)	Cohort	UST vs placebo	confirmed surgery with available operative or procedure notes.	5 yrs	NR	NR	HR	0.31

anti-TNF: anti–tumor necrosis factor, ctrl: control, exp: experimental, HR: hazard ratio, IFX: infliximab, mo: months, NR: not reported, UST: ustekinumab, VDZ: vedolizumab, yrs: years

In Shani et al., the anti-TNF comparator consisted of infliximab and adalimumab (pooled analysis).
In Gubatan et al., anti-TNF therapy included infliximab, adalimumab, and certolizumab (pooled analysis).

Supplementary Table S8: Relapse Rates Across Advanced Therapies for Perianal Crohn's Disease.

Author (year)	Design	Therapy (Exp vs Ctrl)	Definition Used	Follow-up	Exp n/N (%)	Ctrl n/N (%)	Effect Measure	p-value
Chen et al., (2025)	Cohort	UST vs IFX	The appearance of drainage through fistulas that were previously in remission and/or increasing luminal activity	For UST: 10.1 mo<br>For IFX: 31 mo	10/49 (20.4)	28/48 (58.3)	NR	<0.001
Shani et al., (2025)	Cohort	VDZ vs anti-TNF	the onset of symptoms or observable signs of active perianal fistula in previously asymptomatic individuals, as determined by the treating physician, or the diagnosis of a new perianal fistula, confirmed either clinically or via MRI.	6 mo	8/33 (24.2)	8/33 (24.2)	NR	0.63
Shani et al., (2025)	Cohort	UST vs anti-TNF	the onset of symptoms or observable signs of active perianal fistula in previously asymptomatic individuals, as determined by the treating physician, or the diagnosis of a new perianal fistula, confirmed either clinically or via MRI.	6 mo	10/33 (34.5)	8/33 (24.2)	NR	0.63
Gubatan et al., (2023)	Cohort	Anti-TNF vs placebo	perianal abscess recurrence were determined through provider surgical (EUA) notes and imaging results.	5 yrs	NR	NR	HR	<0.001
Gubatan et al., (2023)	Cohort	VDZ vs placebo	perianal abscess recurrence were determined through provider surgical (EUA) notes and imaging results.	5 yrs	NR	NR	HR	0.24

Gubatan et al., (2023)	Cohort	UST vs placebo	perianal abscess recurrence were determined through provider surgical (EUA) notes and imaging results.	5 yrs	NR	NR	HR	<0.01

anti-TNF: anti–tumor necrosis factor, ctrl: control, exp: experimental, HR: hazard ratio, IFX: infliximab, mo: months, NR: not reported, UST: ustekinumab, VDZ: vedolizumab, yrs: years

In Shani et al., the anti-TNF comparator consisted of infliximab and adalimumab (pooled analysis).
In Gubatan et al., anti-TNF therapy included infliximab, adalimumab, and certolizumab (pooled analysis).

Supplementary Table S9: CRP Change Across Advanced Therapies for Perianal Crohn's Disease.

**Author (year)**	**Design**	**Therapy (Exp vs Ctrl)**	**Definition Used**	**Follow-up**	**Exp n/N (%) Or Median change [IQR]**	**Ctrl n/N (%) Or Median change [IQR]**	**Effect Measure**	**p-value**
Chen et al., (2025)	Cohort	UST vs IFX	a CRP level of < 5 mg/L according to the center's inspection standard	For UST: 10.1 mo<br>For IFX: 31 mo	31/49 (63.3)	35/48 (72.9)	NR	0.313
Reinisch et al.., (2024)	RCT	200 mg filgotinib vs placebo	reductions in CRP levels from baseline to Week 10	24 wks	− 1.3 mg/L [−7.5, 0.6]	− 0.1 mg/L [−6.4, 2.4]	NR	NR

ctrl: control, exp: experimental, IFX: infliximab, IQR: interquartile mo: months, NR: not reported, RCT: randomized clinical trial, UST: ustekinumab, yrs: years

Supplementary Table S10: FCP Change Across Advanced Therapies for Perianal Crohn's Disease.

Author (year)	Design	Therapy (Exp vs Ctrl)	Definition Used	Follow-up	Exp median change [IQR]	Ctrl median change [IQR]	Effect Measure	p-value
Reinisch et al.., (2024)	RCT	200 mg filgotinib vs placebo	Change in fecal calprotectin from baseline to week 24	24 wks	-2 [-492,176]	38 [0, 118]	NR	NR
Reinisch et al.., (2024)	RCT	100 mg filgotinib vs placebo	Change in fecal calprotectin frombaseline to week 24	24 wks	-12 [-166,195]	38 [0, 118]	NR	NR

ctrl: control, exp: experimental, IQR: interquartile range, NR: not reported, RCT: randomized clinical trial

Supplementary Table S11: Subjective Outcome Across Advanced Therapies for Perianal Crohn's Disease.

**Author (year)**	**Design**	**Therapy (Exp vs Ctrl)**	**Definition Used**	**Follow-up**	**Mean ± SD**	**Mean ± SD**	**Effect Measure**	**p-value**
Reinisch et al.., (2024)	RCT	200 mg filgotinib vs placebo	11-point NRS scores for perianal pain at week 24	24 wks	− 3.4 ± 3.1	-1.3 ± 1.8	NR	NR
Reinisch et al.., (2024)	RCT	100 mg filgotinib vs placebo	11-point NRS scores for perianal pain at week 24	24 wks	− 1.8 ± 3.1	-1.3 ± 1.8	NR	NR
Hong et al., (2020)	Cohort	IFX vs placebo	Chinese version of the SIBDQ.	6–12 mo	2.6 ± 3.9	0.7 ± 3.2	NR	0.015

ctrl: control, exp: experimental, IFX: infliximab, mo: months, NR: not reported, SD: standard deviation, wks: weeks, RCT: randomized control trial
